# Effectiveness of neonatal early supported transfer to home interventions: implications for practise

**DOI:** 10.55975/FZHP6510

**Published:** 2023-05-01

**Authors:** Natalie Morgan, Oluwaseun Helen Ibiwoye, Candiss Argent, James Hill, Oliver Hamer

**Affiliations:** NHS Royal Preston Hospital; NIHR Applied Research Collaboration - Northwest Coast (ARC-NWC); Lancashire Teaching Hospitals NHS Trust; University of Central Lancashire; University of Central Lancashire

## Abstract

In the UK, more than 38,000 preterm infants are admitted to Neonatal Intensive Care Units every year. NICU stays, along with perceived vulnerability, have been shown to increase parental stress. Parental stress at this stage of infant development has been associated with a deterioration of the long-term parent-infant relationship. Parental stress may be reduced by early educational, behavioural, and psychological support interventions. However, there is a dearth of literature that has synthesised whether these early discharge (supported transfer to home) interventions are clinically effective. This commentary aims to critically appraise a recent systematic review by Hamer et al, 2022 and expand upon the implications of the findings for clinical practice.

## Introduction

In the UK, more than 38,000 preterm infants (less than 37 weeks’ gestation) are admitted to Neonatal Intensive Care Units (NICU) every year.^1^ It is well established that preterm infants admitted to NICU require careful monitoring due to the increased risks of complications.^2^ This increased monitoring, along with perceived vulnerability (and the environment of a NICU), have been shown to increase parental stress.^3^ Parental stress is intensified by prolonged separation from the preterm infant which often leads to frequent misunderstanding of behavioural changes, adversely affecting the long-term parent-infant relationship.^4^ Parental stress as a consequence of long NICU stays has also been shown to be detrimental to the future development of the infant.^5^ Parental stress may be reduced by early educational, behavioural, and psychological support interventions.^6^

The Neonatal Early Supported Transfer to Home approach is the concept of transition of care to home.^7^ Early supported discharge to home (earlier than standard care), is the wish of many parents who feel overwhelmed by the NICU.^8^ Early supported discharge to home allows empowerment of parents to have greater involvement in the care and progression of the infant ^9^

There is currently limited funding for continuing care in a community setting to facilitate early supported discharge from NICUs.^10^ The Neonatal Early Supported Transfer to Home approach is yet to be extensively evaluated for clinical effectiveness.^11^ Research is now needed to be establish whether this approach would outweigh possible adverse outcomes such as post discharge parental stress or rehospitalisation of the preterm infant. A recent systematic review by Hamer et al, 2022 assessed the current evidence of neonatal early supported transfer to home interventions.^12^

This commentary aims to critically appraise the methods used within the review by Hamer et al, 2022, evaluate the current evidence, and expand on its implications for clinical practice.

## Methods of the review by Hamer et al, 2022

A comprehensive search was undertaken on six databases; CENTRAL, CINAHL, EMBASE, MEDLINE, PsychInfo, and the World Health Organisation International Clinical Trials Registry Platform (searched from inception to February 2022). Only randomised or nonrandomised controlled trials and observational studies including parents or primary caregivers of preterm infants who were involved in early supported transfer interventions involving early discharge planning, education, or training (which allows them to continue their progress at home) were included. The included studies needed to have a control group of either usual care or different types of early discharge interventions. Studies that were not in English were excluded. The primary outcomes were duration of NICU stay and hospital re-admission rates. The secondary outcomes were parental stress, parental well-being, parental confidence, infant weight gain and breastfeeding. The included studies were summarised narratively and where data was available, meta-analysis using a random effects model was employed to synthesis data.^12^

The titles, abstract and full texts of the search results were independently screened by two reviewers. Data extraction was performed by three reviewers independently. A fourth reviewer checked the data extractions, and a fifth reviewer resolved any disagreements. Risk of bias was independently assessed by two reviewers and a third reviewer verified the assessments. The Cochrane risk-of-bias 2 tool was used for RCTs (at study level), and the Risk of Bias in Non-Randomised Studies of Intervention (ROBINS-1) tool was used for non-randomised studies.^13^.

## Results

The systematic review included 10 studies involving 12821 participants. Studies were conducted in seven countries. All studies were conducted in hospital settings and ranged in duration from 12 months to 12 years. The included studies were observational studies (n = 5), non-randomised control studies (n = 2) and randomised controlled trials (n = 2). Preterm infant participants age ranged from 29–35 weeks. Interventions varied across the studies but typically aimed to reduce the length of hospital stay, improve parents’ preparedness to take their infant home, and teach parents about caretaking of their child.

Interventions were delivered by a range of professionals including neonatology, home-care nurses, research nurses, primary care paediatricians, programme managers or members of outreach teams. The interventions had several components but largely included home visits, educational sessions, and other support (e.g., telephone support, takeaway information, parental visits etc).^12^

### Hospital stays

A meta-analysis using a random effects model showed that early supported transfer home enabled preterm infants to be discharged 10.4 days earlier compared to those receiving standard care (four studies: minus 10.4 days, 95% CI -13.8 to -7.1, P = < 0.001, RoB = non-RCT: three serious & one moderate concerns).

### Hospital re-admissions

Meta analysis using a random effects model showed that there was no evidence of difference in risk between standard care and intervention groups related to hospital re-admissions (three studies: RR 0.91, 95% CI 0.65 to 1.26 P= 0.57, RoB = RCT’s: two some concerns, non-RCT: two serious & two moderate concerns).

### Parental wellbeing

No significant difference in general anxiety, anxiety related to the care of the infant, mental imbalance or well-being scores reported between early supported discharge and the standard care groups (two studies: at one year follow up; P= >0.05, RoB = RCT: one some concerns, non-RCT: one serious).

### Parental confidence

No significant difference in parental confidence scores were observed between early supported discharge and the standard care groups (two studies: measurements at baseline, discharge, home or one year follow up; P= >0.05, RoB = non-RCT: one serious, one moderate).

### Infant weight gain

Meta analysis using a random effects model indicated that there was no evidence of difference observed in weight gain of preterm infants between early supported discharge intervention compared to those who received standard care (three studies: Mean difference= 1.150 grams per day, 95% CI: -1.85 to 4.15, P= 0.454, RoB = RCT: one some concerns, non-RCT: two serious).

### Breastfeeding

No significant difference in rates of exclusive infant breastfeeding, rates of partial infant breastfeeding or duration of breastfeeding were observed between early supported discharge and standard care groups (five studies: at three weeks, six weeks, or six-month follow-up; P= >0.05, RoB = RCT: one some concerns, non- RCT: one serious, three moderate)

## Commentary

The AMSTAR-2 critical appraisal tool for systematic reviews was employed to appraise the review.^14^ Of the 16 criteria, 14 were met, indicative of a robust and comprehensive summary. Two criteria were not met as there was no assessment of publication bias and the RoB2 assessment tool was conducted at a study level, not at a outcome level.^15^

Findings of the review suggest that early supported transfer home interventions may reduce hospital stay of babies (born before 37 weeks gestation) by up to 10 days (compared to routine NICU care), without increasing hospital readmission rates, worsening parental well-being, or impacting on infant weight gain or breastfeeding.^16^ The review synthesised that early supported transfer home interventions may improve infant-parent bonding and could have cost-saving implications for healthcare services (may save up to eight thousand pound per infant compared to usual NICU care).^17^

### Implication for practise – midwifery and nursing

In terms of clinical practise, the review identified consistent components of early supported transfer to home interventions.^12^ The synthesis of evidence suggests that components of education, home visits, takeaway information, telephone support, education sessions and programme management are needed for early supported transfer to home interventions.^12^

Although the results of the review need to be interpreted with caution, the findings suggest that preterm babies may be able to safely go home earlier than previously permitted.^12^ That said, the home environment needs to be appropriate and safe for families to be considered for early supported transfer interventions.^18^ In order to determine this, clinicians would need to conduct a home visits and assess its suitability against specific criteria, for example the home needs to be adequately warm (consistently around 18 degrees) to support the infant’s thermal environment without recourse to excessive clothing or bedding.^18^ These criteria may pose a challenge to parents in low-income households who cannot afford the costs of caring for a preterm infant at home.^19^ These considerations are key for implementation, as healthcare professionals need to be alerted to the possible impact health inequalities may have on the success of these interventions.

Additional to these considerations, changes in staff dynamic and available resources may also be integral to the successful implementation of the intervention (in view of the anticipated increased number of babies being transferred home). Key challenges for implementation surround funding, which may be aided by staff to baby ratio as they are less intensive within community settings.^20^ Additional challenges include staff attitude towards the intervention, relating to an infant’s readiness for transfer home.^21^ Comprehensive staff education and training may alleviate some of these challenges and bring about a change in attitude.^24^

Implementation of the intervention would benefit from ‘Agenda for Change’ nurses, midwives (bands 4 to 6) and a consultant neonatologist that can provide swift safety escalation mechanisms when needed.^22^ Further to this, every effort should be made to safeguard vulnerable children by conducting risk assessments before the decision to transfer infants home is made.^21^ Provision of training, education and practical resources such as equipment required for monitoring the babies’ weight, thermoregulation, and nasogastric feeding should be provided to facilitate the intervention within the home. Policies for lone working should also be developed to mitigate safety risks for midwives working in the community.

Although there are some implications for future practise, recommendations for implementation into clinical practice cannot yet be made due to the lack of high-quality evidence. Further research involving high quality RCT’s assessing the effectiveness of early supported transfer home interventions on key clinical and psychological outcomes are required. Further studies should address methodological limitations, adopting high quality approaches which include randomisation and follow up (short and long term) to minimise bias. However, there is acknowledgement that inclusion rates are typically low within individual settings which makes randomisation logistically challenging.

In addition to improving methodological quality, studies should consistently report key outcomes such as hospital re-admission rates, parental wellbeing, and parental stress to determine the wider effect of early supported transfer to home interventions for parents and preterm infants. Studies would benefit from the adoption of a core outcome set (COS) which may standardise outcome selection in future studies, and ensure they are relevant to those affected by neonatal intensive care.

### Practise challenge questions

1. What are the limitations and strengths of the evidence synthesised by the systematic review?
2. What are the practical considerations when implementing an early supported transfer to home intervention?
3. What may be some of the challenges of implementing an early supported transfer to home intervention?
4. What evidence-based components should be included in an early supported transfer to home intervention?

## Key Points

- Early supported transfer home interventions may reduce hospital stay without affecting hospital readmission rates, parental well-being, babies weight gain and breastfeeding.
- The review indicates that early supported transfer home interventions may improve infant-parent bonding and could have cost-saving implications for healthcare services.
- Recommendations for implementation into clinical practice cannot yet be made due to the lack of high-quality evidence.
- Further high-quality research is needed to assess the effectiveness of early supported transfer home interventions on key clinical and psychological outcomes
